# Immediate Effects of Acceptance and Commitment Therapy in Children with ADHD: A Pilot Resting-State fNIRS Study

**DOI:** 10.3390/brainsci16060564

**Published:** 2026-05-27

**Authors:** Betül Koçarslan, Herdem Aslan-Genç, Emre Yorgancıgil, Gülnaz Yükselen, Zeynep Seda Albayrak, Gökçen Aydın, Sinem Burcu Erdoğan, Ata Akın, Asli Demirtas-Tatlidede

**Affiliations:** 1 Department of Neuroscience, Bahçeşehir University, İstanbul 34734, Türkiye; betul.kocarslan@bahcesehir.edu.tr; 2 Department of Child and Adolescent Psychiatry, School of Medicine, Koç University, İstanbul 34010, Türkiye; heaslan@ku.edu.tr (H.A.-G.); zalbayrak@ku.edu.tr (Z.S.A.); 3 Department of Biomedical Engineering, Faculty of Engineering and Natural Sciences, Acıbadem Mehmet Ali Aydınlar University, İstanbul 34752, Türkiye; emre.yorgancigil@acibadem.edu.tr (E.Y.); gulnaz.yukselen@acibadem.edu.tr (G.Y.); sinem.erdogan@acibadem.edu.tr (S.B.E.); 4 Department of Psychological Counseling and Guidance, TED University, Ankara 06420, Türkiye; gokcen.aydin@tedu.edu.tr; 5 Department of Neurology, Faculty of Medicine, Bahçeşehir University, İstanbul 34734, Türkiye

**Keywords:** Attention-Deficit/Hyperactivity Disorder (ADHD), Functional Near-Infrared Spectroscopy (fNIRS), Acceptance and Commitment Therapy (ACT), emotion regulation, children, prefrontal cortex, resting state, hemodynamic response

## Abstract

**Background:** Children with ADHD exhibit deficits not only with attentional control but also with emotional dysregulation and experiential avoidance. Acceptance and Commitment Therapy (ACT) directly targets these processes by enhancing psychological flexibility and reducing maladaptive responses to internal experiences. Functional Near-Infrared Spectroscopy (fNIRS) offers a feasible and reliable method for assessing cortical hemodynamics in the ADHD population due to its portability and robustness to motion. This study aimed to investigate the immediate effects of a single-session ACT intervention on resting-state prefrontal hemodynamic activity in children with ADHD. **Methods:** Twenty children with ADHD underwent a single session of emotion regulation-focused ACT intervention. Resting-state fNIRS data were acquired immediately before and after the intervention. Changes in oxygenated (HbO) and deoxygenated hemoglobin (HbR) concentrations were statistically compared using paired *t*-tests. **Results:** All participants completed the protocol, and fNIRS acquisition was well tolerated. Post-intervention analyses revealed significant hemodynamic alterations after the session, marked by increased HbO at 0–240 s in the right lateral prefrontal cortex. HbO levels after the intervention were associated with SNAP hyperactivity scores. **Conclusions:** These findings provide preliminary evidence that single-session ACT intervention may be associated with short-term changes in resting hemodynamic activity in children with ADHD. fNIRS may demonstrate utility as a sensitive modality for detecting short-term intervention-related changes in neural activity. Given the exploratory nature of the study, future research with larger, controlled, and longitudinal designs is needed to evaluate the reproducibility of these findings and the clinical relevance of the observed hemodynamic changes.

## 1. Introduction

Attention Deficit Hyperactivity Disorder (ADHD) is one of the most common neurodevelopmental disorders affecting approximately 5–7% of children and adolescents [1]. Beyond the core symptoms of inattention, hyperactivity, and impulsivity, growing evidence suggests that deficits in emotion regulation (ER) constitute a central feature of ADHD [2,3]. Emotion regulation has been defined as the processes by which individuals control their emotional responses in order to guide their behavior in line with situational demands and both short-term and long-term goals in every context [4,5,6].

The conceptualization of ADHD as a disorder of dispersed functional networks has gained support in recent years [7]. Abnormalities between or within large-scale networks are thought to underlie the cognitive and affective deficits exhibited in ADHD [8,9]. To identify the abnormalities of functional networks in ADHD and the interactions between them, resting-state functional connectivity (rsFC) techniques have been widely used and particularly highlighted disruptions in the interaction between the default mode network (DMN) and the frontoparietal network (FPN) [10,11,12,13,14,15]. Within this framework, the DMN—anchored in medial prefrontal regions—is associated with internally oriented and self-referential processes, whereas the FPN, including the lateral prefrontal cortex (PFC), supports top-down executive control and regulation [16,17]. In ADHD, reduced connectivity between executive attention and affective circuitry and diminished engagement of executive control systems have been consistently reported, contributing to impairments in emotion regulation and increased susceptibility to distraction, particularly in emotionally salient contexts [18,19,20].

From an emotion regulation perspective, effective functioning depends on the dynamic balance between bottom-up emotion generation from limbic structures and top-down regulation supported by the PFC. Notably, both the DMN and FPN overlap with neural systems supporting executive functioning and emotion regulation. In ADHD, evidence points to reduced within-network connectivity in the DMN, suggesting impaired integration of internal mental states [12,21,22,23,24]. Further, the dorsolateral prefrontal cortex (DLPFC) and VLPFC [25,26,27] show considerable evidence of hypoactivity in ADHD [13,18,23,26,27,28,29,30,31]. These regions contribute to the FPN [32] and are critical for cognitive control processes, including sustained attention, inhibition, working memory, and goal-oriented decision-making [16,33]. The alterations in these regions indicate a dysfunction in prefrontal regulatory mechanisms and their coordination with internally oriented networks in ADHD.

Mindfulness-based therapies have been reported to alleviate ADHD symptoms and to support the self-regulation of emotions and attention [34,35]. Acceptance and Commitment therapy (ACT) is a third-wave behavioral intervention that integrates mindfulness and acceptance-based processes, including mindfulness [36], that has demonstrated efficacy across a range of psychiatric disorders, such as depression, anxiety, and core ADHD symptoms such as hyperactivity, inattention, emotional problems, academic procrastination, and self-regulation [37,38,39,40,41,42,43]. ACT promotes awareness of internal experiences and external stimuli [44,45]. Rather than suppressing internal experiences, ACT emphasizes the development of psychological flexibility through mindful awareness, which may involve the simultaneous engagement of self-referential (DMN) and cognitive control (FPN) processes. Instead of changing the content of patients’ unwanted thoughts, feelings, and emotions, ACT aims to change how patients interact with them [36]. Children with ADHD who complete ACT training become better at expressing and managing even their unpleasant emotions in various settings [46]. While standard ACT training spans between 6 and 8 weeks, several studies revealed that participants reported significant improvements in dealing with difficult emotions even after single-session ACT interventions [47,48,49].

Neuroimaging techniques have been widely used to elucidate the neural mechanisms underlying ADHD. Among these, Functional Near-Infrared Spectroscopy (fNIRS) is an optical and noninvasive neuroimaging technique measuring concentration changes in both oxyhemoglobin (HbO) and deoxyhemoglobin (HbR), providing an indirect index of cortical activity. Similar to functional magnetic resonance imaging (fMRI), fNIRS captures hemodynamic responses associated with neural activation and is particularly well suited for pediatric populations due to its low cost, low sensitivity to motion artifacts, ecological validity, and portability [50]. A substantial body of fNIRS research has advanced our understanding of the neurobiology of ADHD, primarily focusing on task-related activities such as attention [51], inhibition [52], cognitive flexibility [53], and working memory [54]. More recent investigations have further extended this evidence base to encompass hemodynamic markers of pharmacological response in pediatric ADHD [55] and machine-learning-based identification of ADHD from prefrontal hemodynamic signatures [56]. However, relatively few studies have examined the resting-state changes associated with therapeutic interventions or whether such treatments can remediate aberrant resting-state brain activity in ADHD [57]. This gap is particularly relevant for psychotherapeutic interventions such as ACT and other mindfulness-based approaches, whose proposed neural mechanisms involve enhanced prefrontal regulation of limbic reactivity, increased meta-awareness mediated by medial and dorsolateral prefrontal networks, and modulation of default-mode and salience-network engagement [58,59,60]. ACT and mindfulness practices may also strengthen top-down prefrontal regulation and reorganize the self-referential processing [60]. In light of the previous studies, resting-state prefrontal fNIRS recordings may be a suitable method for capturing immediate, neural correlates of brief ACT delivery in children with ADHD.

Within this context, examining prefrontal activity using fNIRS offers a targeted approach to capture rapid functional hemodynamic changes in major cortical hubs, particularly in the lateral and medial prefrontal regions, which are accessible to fNIRS measurements. Understanding whether ACT modulates hemodynamic and neural activity in these regions may provide important insights into how therapeutic processes influence the balance between internally oriented processing and top-down control in children with ADHD. To our knowledge, no previous study has yet examined the immediate hemodynamic effects of a single-session ACT intervention on resting-state activity in children with ADHD using fNIRS. Hence, the primary aim of this study was to explore whether a single-session ACT intervention may be associated with changes in prefrontal activity, as measured by resting-state fNIRS, in children with ADHD. As a secondary aim, we sought to explore the associations between the clinical measures and the hemodynamic changes in prefrontal activation following the ACT session.

## 2. Materials and Methods

### 2.1. Participants

This exploratory study employed a within-subject pre-post intervention design. The participant group consisted of 20 children who met DSM-5 criteria for a clinical diagnosis of ADHD, aged 9 to 13 years. Participants were recruited from the outpatient unit of Koç University Hospital, Department of Child and Adolescent Psychiatry.

Inclusion criteria comprised right-handedness, having a symptom severity score of three or greater on the Clinical Global Impression Scale (CGI), and an intelligence quotient of 85 or above on the Wechsler Intelligence Scale for Children-IV (WISC-IV). The exclusion criteria include prior head trauma, diagnosis of depression or anxiety disorders, developmental, psychiatric, or neurological disorders such as Autism Spectrum Disorder (ASD), learning disabilities, Tourette Syndrome, mood disorders, psychotic disorders, chronic medical illnesses, and the use of pharmacological treatments unrelated to ADHD. All participants met the DSM-5 criteria for ADHD, as confirmed by a licensed child and adolescent psychiatrist. The diagnosis was verified through the administration of the K-SADS-5 semi-structured interview. Participants were not drug-naïve; those receiving psychostimulant medication were instructed to discontinue medication at least 24 h prior to the fNIRS assessment to minimize acute medication effects on hemodynamic responses.

Ethics committee approval for the study was obtained from the Ethics Committee of Acıbadem University (2022-20/14, approval date 30 December 2022). Written informed consent was obtained from all participants and their guardians, and assent was obtained from all children prior to participation. The study was conducted in accordance with the Declaration of Helsinki.

### 2.2. Clinical Measures

At baseline, all participants and their caregivers completed a battery of standardized measures designed to capture psychological flexibility, emotional and behavioral symptoms, and functional impairment. Attention was evaluated using the Conners Continuous Performance Test [61], a standardized measure of sustained attention and inhibitory control.

#### 2.2.1. Child and Adolescent Mindfulness Measure (CAMM)

The Child and Adolescent Mindfulness Measure (CAMM) was used to assess mindfulness levels in children. It is an 8-item self-report scale developed by Greco, Baer, and Smith [62] that evaluates present-moment awareness and nonjudgmental, nonavoidant reactions to thoughts and emotions. The Turkish adaptation was conducted by Çıkrıkçı [63]. Items are rated on a 5-point Likert scale ranging from 0 (never true) to 4 (always true), with all items reverse scored. Higher scores indicate greater levels of mindfulness.

#### 2.2.2. Avoidance and Fusion Questionnaire-Youth 8 (AFQ-Y8)

The Acceptance and Fusion Questionnaire (AFQ-Y8) was used to assess psychological flexibility. It is an 8-item scale self-report measure for children and adolescents aged 9 to 17 years [64]. The Turkish version was validated by Büyüköksüz and Erözkan [65]. Items are rated on a 5-point Likert scale ranging from 0 (not at all true) to 4 (very true). Higher scores indicate greater psychological inflexibility (i.e., lower psychological flexibility).

#### 2.2.3. Children’s Depression Inventory (CDI)

The Children’s Depression Inventory (CDI) was used to assess depressive symptoms in children. Developed by Kovacs [66] and adapted to Turkish by Öy [67], the CDI is a 27-item self-report measure assessing symptoms such as low mood, anhedonia, suicidal ideation, and disturbances in sleep and appetite. Each item consists of three response options scored from 0 to 2, reflecting increasing severity. Higher scores indicate greater severity of depressive symptoms.

#### 2.2.4. Screen for Child Anxiety Related Emotional Disorders (SCARED)

The Screen for Child Anxiety Related Emotional Disorders (SCARED) was used to assess anxiety symptoms [68]. The Turkish reliability and validity study was conducted by Çakmakçı [69]. The scale consists of 41 items rated on a 3-point Likert scale ranging from 0 (not true or hardly ever true) to 2 (very true or often true) and includes five subscales: panic/somatic symptoms, school phobia, social anxiety, separation anxiety, and generalized anxiety. Higher scores indicate greater levels of anxiety symptoms.

#### 2.2.5. WEISS Functional Impairment Rating Scale

The Weiss Functional Impairment Rating Scale (WFIRS) was used to assess functional impairment in daily life. Developed by Weiss [70], the Turkish version was validated by Tarakçıoğlu et al. [71]. The scale consists of 44 items rated on a 4-point Likert scale ranging from 0 (never/not at all) to 3 (very often/very much). Higher scores indicate greater functional impairment.

#### 2.2.6. SNAP-IV ADHD Screening Form

The Swanson, Nolan, and Pelham Rating Scale–Version IV (SNAP-IV) was used to assess ADHD symptom severity according to parent reporting [72]. The scale consists of 18 items corresponding to the two core symptom domains of ADHD: inattention (9 items) and hyperactivity/impulsivity (9 items). Items are rated on a 4-point Likert scale ranging from 0 (not at all) to 3 (very much). Higher scores indicate greater ADHD symptom severity.

#### 2.2.7. Conners Continuous Performance Test (CPT-3)

CPT-3 is a computerized test used to determine the level of attention and cognitive abilities in individuals aged 8 and older. As a continuation of previous Conners attention tests [73], CPT-3 [61] was an updated measure of attention and inhibition processes. Participants are instructed to respond to all letters displayed on the screen except the ‘X’. The administration time of CPT-3 lasts approximately 14 min. The test provides an insight into the level of participants’ attentiveness, impulsivity, and vigilance.

### 2.3. Acceptance and Commitment Therapy Intervention (ACT)

Acceptance and Commitment Therapy (ACT) intervention was administered immediately following the fNIRS assessment. The intervention was a brief, structured psychoeducational session grounded in the principles of ACT, with a focus on enhancing psychological flexibility in children with ADHD through a range of experiential exercises, which could subsequently be generalized to real-life situations in which the participant experiences difficulties [36]. The single-session intervention protocol was developed based on existing literature about ACT approaches for ADHD. Specifically, the session structure and content were designed mainly based on the previous ADHD studies [43,74,75,76]. Drawing on these sources, a developmentally appropriate protocol was adapted for children with ADHD by simplifying core ACT processes (e.g., present-moment awareness, values clarification, acceptance, and committed action) and integrating age-appropriate experiential exercises, metaphors, and behavioral activities. The session content is presented in Table 1. The session lasted approximately 20 min and was delivered by a psychologist (B.K.) who had received formal training in ACT and performed the ACT intervention under the supervision of a psychological counselor (G.A.) who has a specialty in ACT. The session followed a predefined structure with consistent components and sequence across participants. The session consisted of four sequential components.

#### 2.3.1. Emotional Awareness and Psychoeducation (5 min)

The session began with an emotion identification exercise using visual emotion cards. Participants were asked to identify which emotions they recognized and to describe situations in which they experienced these emotions. This activity aimed to enhance emotional awareness and labeling skills. Following this, a brief psychoeducational component was introduced to explain the function of the mind. Using a developmentally appropriate “caveman mind metaphor,” the therapist explained that the human mind evolved to detect danger and protect the individual. This was then linked to modern-day experiences, illustrating how the mind may generate distressing thoughts even in the absence of real danger. This component targeted awareness and acceptance of internal experiences.

#### 2.3.2. Present-Moment Awareness (5 min)

The second component was mindfulness practices, where participants were guided to notice how the mind often shifts between past and future-oriented thoughts. A brief mindfulness exercise was then conducted using a “jellybean exercise,” in which participants were instructed to focus on the sensory properties (e.g., texture, taste, smell) of the object in the present moment. This exercise aimed to foster present-moment awareness.

#### 2.3.3. Values Clarification (5 min)

After the mindfulness exercise, values clarification was introduced via an exercise called “lifetime achievement award”. Participants were asked to imagine receiving an award and to reflect on how they would like to be described by others. A child-friendly values list was then provided, and participants selected values that were personally meaningful to them. This component targeted the identification of values.

#### 2.3.4. Acceptance and Committed Action (5 min)

The therapist discussed how acting in line with one’s values is not always easy and may involve encountering difficult thoughts and emotions. This component was presented to the participant in line with the exercise ‘The Winding and Rocky Road of Values: Dealing With Hooks’ [77]. Participants were encouraged to consider small, concrete actions they could take in alignment with their chosen values, despite potential internal discomfort. This component targeted acceptance and committed action processes.

### 2.4. Functional Near-Infrared Spectroscopy (fNIRS)

After the completion of the scales and CPT-3 administration, the participant was taken to an adjacent room and introduced to the study procedures. Participants were familiarized with the fNIRS equipment and were allowed to examine the fNIRS cap and touch the infrared optodes to increase comfort. Any questions or concerns were addressed before the measurement began. The cap was placed over the prefrontal cortex of the participant, with 8 light sources and 8 detectors in accordance with the International 10–20 system. Participants were seated comfortably while the cap was placed and were asked to remain still while the fNIRS channels were optimized. After the optimization process, participants were briefly shown their brain activity on the screen. The record begins with the participant being instructed to look at the blank computer screen during the recording while sitting still and keeping their eyes open.

Participants sat in a quiet room while the hemodynamic changes in the individuals’ frontal cortex were measured using NIRSport System fNIRS (NIRx Medical Technologies, LLC, Berlin, Germany). Changes in oxygenated hemoglobin (HbO) and deoxyhemoglobin (HbR) concentrations during an 8-min resting state were measured. The neural activity was recorded immediately before the ACT intervention. After the single-session ACT intervention, a second resting-state fNIRS recording was conducted (post-intervention).

#### 2.4.1. Data Preprocessing

All signal processing and analysis were conducted using custom-written scripts in MATLAB program (Version 2022b). Raw intensity light data were preprocessed using Homer-3 functions integrated within a custom MATLAB pipeline. At the first step, raw intensity measurements were converted to changes in optical density (ΔOD) using the hmrR_Intensity2OD function. Channels exhibiting insufficient signal quality (identified by non-finite or non-positive intensity values) were detected and excluded through an automated channel-pruning procedure prior to raw intensity conversion. The ΔOD data were then transformed into relative concentration changes of oxygenated hemoglobin (HbO) and deoxygenated hemoglobin (HbR) with the modified Beer–Lambert law (hmrR_OD2Conc) with partial pathlength factors set to [1.0, 1.0] for the 760 nm and 850 nm wavelengths [78].

A zero-phase bandpass filter was applied with a passband of 0.008–0.08 Hz (hmrR_BandpassFilt). The high-pass cutoff at 0.008 Hz removed very slow instrumental drifts and baseline wander, while the low-pass cutoff at 0.08 Hz was specifically chosen to preserve spontaneous low-frequency oscillations (LFOs) associated with resting-state cerebral autoregulation and neurovascular coupling, which are predominantly observed below 0.1 Hz in pediatric populations [79]. Our 0.008–0.08 Hz passband corresponds to the canonical frequency range specifically established by resting-state fNIRS analysis. Band-limiting hemodynamic signals to 0.009–0.08 Hz isolates the neurogenic component of spontaneous cortical activity from systemic confounds and has since been adopted as a methodological standard for non-task fNIRS recordings [80]. Current bandpass filter effectively attenuates cardiac pulsation (~1 Hz), respiration (~0.2–0.4 Hz), and Mayer wave oscillations (~0.1 Hz), which are the major sources of systemic physiological noise in fNIRS recordings and are particularly prominent in pediatric groups due to higher resting heart and respiratory rates [81]. Superficial signal regression was subsequently performed to mitigate extracerebral contamination originating from hemodynamic fluctuations in the scalp and skull. The mean time course across all eight short-separation channels was computed to form a single global extracerebral regressor. This regressor was removed from each of the 22 long-separation channels via ordinary least-squares regression, applied separately for HbO and HbR. The use of a global short-channel average signal was adopted to provide a more spatially robust estimate of systemic scalp hemodynamics, reducing the sensitivity to localized optode coupling variability that is common in pediatric recordings, where head size variation and participant compliance can introduce inconsistent scalp contact across the probe array [82,83]. As the resting-state protocol involved no active tasks or movements, no explicit motion artifact correction algorithm was applied. Motion artifacts in hemodynamic data predominantly produce high-frequency spikes and abrupt baseline shifts whose spectral content lies largely above the 0.08 Hz low-pass cutoff, which we used. Thus, the applied 0.008–0.08 Hz bandpass filter inherently reduces the majority of motion-related disturbances. Short-channel regression further contributes by removing scalp-level hemodynamic fluctuations, which are mostly modulated by head movement [84]. Our combined bandpass and short-channel regression approach has been shown to provide adequate artifact suppression for continuous rest recordings in cooperative pediatric participants and supports its appropriateness for the present protocol [79,85]. After short-channel regression, 22 cortical long channels remained per chromophore. Total hemoglobin concentration change was computed as the sum of HbO and HbR changes.

#### 2.4.2. Feature Extraction

For regional analysis, two complementary spatial groupings of channels were applied to the 22 long-separation channels based on the optode montage geometry over the prefrontal cortex. The first channel group defined six bilateral ROIs to capture fine-grained lateralized prefrontal organization: Right Dorsolateral, Left Dorsolateral, Right Dorsomedial, Left Dorsomedial, Right Orbitofrontal, and Left Orbitofrontal. The second grouped channels into three broader regions reflecting the lateral–medial functional gradient of the prefrontal cortex: Right Lateral, Medial, and Left Lateral. This coarser parcellation was employed to increase statistical power by pooling a larger number of channels per region and to facilitate group-level comparisons where individual channel variability may obscure broader spatial patterns, particularly in pediatric cohorts where anatomical variability in head size can affect precise channel-to-cortex correspondence. The preprocessed time series of constituent channels of each ROI were averaged to produce a single representative signal per chromophore (HbO and HbR) per region for both spatial grouping schemes.

Two variability-based features, named Range and standard deviation (STD), were extracted from all ROI-averaged signals. These metrics were specifically selected as they are well-suited to characterize spontaneous hemodynamic fluctuations during resting-state conditions where no stimulus-locked averaging or task-evoked response modeling is applicable. In the absence of externally triggered hemodynamic responses, the temporal variability of the fNIRS signal itself becomes the primary measure of interest, reflecting the amplitude and regularity of intrinsic cerebrovascular oscillations [86]. During resting-state fNIRS recordings, where no stimulus onsets exist to anchor conventional general linear model or block-averaged analyses, signal amplitude variability has been established as one of the principal observable hemodynamic quantities indexing spontaneous cortical hemodynamic activity [87,88]. Range is defined as the difference between the maximum and minimum signal amplitude of each preprocessed ROI signal and captures the total excursion of the hemodynamic signal within the defined time interval. It has been used as a marker of cerebrovascular reactivity and autoregulatory capacity during rest conditions [89]. Standard deviation quantifies the overall dispersion of signal fluctuations around the mean and has been widely employed in resting-state fNIRS studies as an index of spontaneous cortical activity magnitude and signal stability [75,79]. From a vital point, both Range and STD features are non-parametric, model-free descriptors that do not assume an underlying canonical hemodynamic response shape. Model-free natures make them inherently appropriate for non-task continuous recordings in which task regressors are unavailable and where alternative spectral or connectivity-based metrics may be confounded by short recording duration or pediatric motion artifacts [87,88]. Both features are particularly appropriate for continuous rest recordings where the signal cannot be decomposed into evoked components from conventional experimental block or event-related averaging [85].

Features were computed over three cumulative time intervals starting from recording onset: 0–120 s, 0–240 s, and 0–480 s. This cumulative windowing approach was adopted to examine whether hemodynamic variability stabilizes or evolves over the course of the rest period, providing insight into the temporal dynamics of spontaneous prefrontal activity. These features were computed separately for the before-experiment and after-experiment rest recordings under both spatial grouping schemes, enabling within-subject comparison of spontaneous hemodynamic variability and its potential modulation by the intervening experimental protocol.

### 2.5. Statistical Analysis

Statistical analyses were conducted using SPSS 29. Descriptive statistics were computed for all continuous variables. Frequencies and percentages were reported for categorical variables, including ADHD subtype and clinical severity levels. The normality of the distributions was assessed using the Shapiro–Wilk test; as the assumption of normality was met, parametric statistical tests were applied.

To examine the effects of the ACT intervention, pre- and post-intervention differences in hemodynamic parameters (i.e., range and Standard deviation) responses (HbO and HbR) in prefrontal regions were analyzed using paired samples *t*-tests. All tests were two-tailed, with a significance level set at *p* < 0.05. To control for multiple comparisons, the Bonferroni correction was applied where appropriate. Additionally, effect sizes were estimated using Cohen’s d to quantify the magnitude of pre–post changes, providing a standardized measure of the significance of the intervention effects. In order to investigate the associations between fNIRS-derived HbO and HbR parameters and clinical measures, Spearman’s rank correlation analyses were performed, given the potential for non-linear relationships. To further evaluate the robustness of observed associations, Kendall’s tau correlations were additionally computed. In addition, Cook’s distance was used to identify potentially influential observations, and sensitivity analyses were conducted where applicable to assess the impact of influential data points. Correlation coefficients were interpreted according to standard thresholds for small, moderate, and large effects.

## 3. Results

### 3.1. Patient Demographics

A total of 21 children with ADHD completed the study. Data from one participant was excluded due to poor-quality resting-state fNIRS data, leaving 20 children for analysis. The patient group included 10 males and 10 females, and the mean age was (11.1 ± 1.4). Of the participants, 35% were classified as the inattentive subtype and 65% as the combined subtype. Patient demographics and clinical characteristics by ADHD subtype have been summarized in Table 2.

All participants completed the protocol. fNIRS recordings were successfully obtained for all participants, with no dropouts and no data exclusions due to intolerance or non-compliance. The procedure was well tolerated and no significant side effects were reported.

### 3.2. Effects of ACT Intervention on fNIRS Hemodynamic Responses

Following the single-session ACT intervention, fNIRS recordings demonstrated significant HbO derived parameter concentration changes in the prefrontal cortical regions of children with ADHD. There was a significant increase and consistent stability in HbO in the right lateral prefrontal cortex between time windows of 0–240 s (*p* < 0.05, Cohen’s d = 0.6). Table 3 summarizes statistically significant differences in range parameters derived from oxyhemoglobin (oxyHb) concentration and deoxyhemoglobin (deoxyHb) (*p* < 0.05) levels following ACT. The complete statistical results, including all intervals and channel groups, are provided in Supplementary Material Table S1.

**Figure 1:**
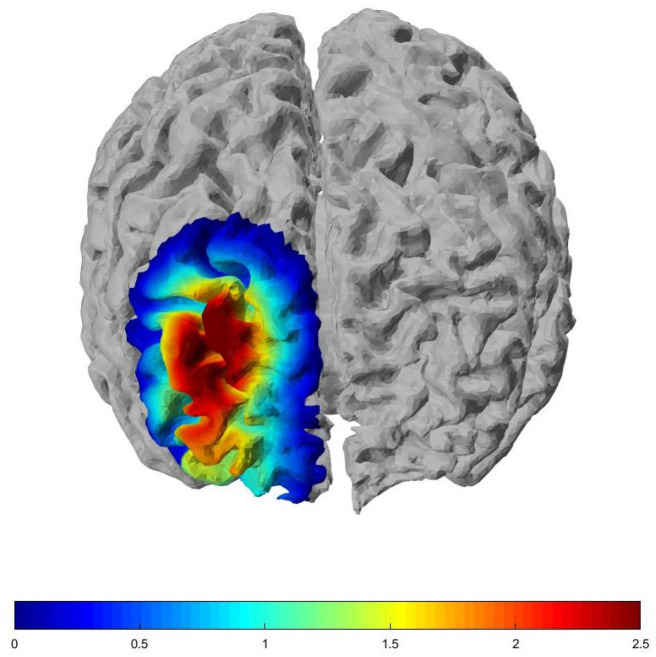
A statistical parameter map depicting hemodynamic differences between post- and pre-intervention conditions (post > pre). Increased HbO in the right lateral prefrontal cortex is shown for the 0–240 s interval. HbO = Oxygenated hemoglobin.

A statistical parameter map illustrating hemodynamic differences between post- and pre-intervention conditions (post > pre) is presented in Figure 1. Thresholded t-statistics obtained from two-tailed *t*-tests on range metrics of the HbO signal are projected onto a brain model. Only the channel showing a statistically significant alteration (*p* < 0.05) is displayed. The t-value computed for the ROI was assigned to the corresponding channel for topographic visualization.

### 3.3. Associations Between Hemodynamic Responses and Clinical Measures

At baseline, HbO levels in right lateral PFC between time windows of 0–480 s showed a significant negative correlation with psychological flexibility (AFQ) (r = −0.484, *p* = 0.049).

**Figure 2:**
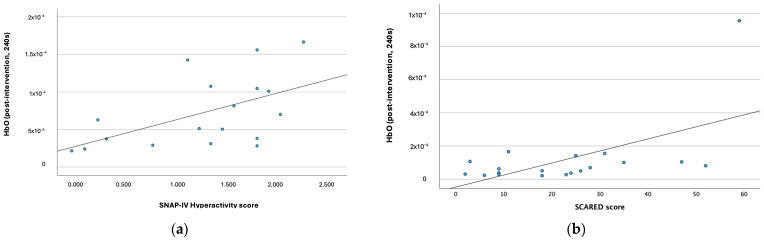
Scatter plots illustrating (**a**) the correlation between SNAP-IV hyperactivity scores and post-intervention HbO levels (0–240 s), (**b**) the correlation between SCARED anxiety scores and post-intervention HbO levels (0–240 s). The solid line represents the linear regression fit.

Post-intervention HbO levels were positively associated with clinical measures (Figure 2). Specifically, post-intervention HbO levels were significantly correlated with hyperactivity scores (SNAP-Hyperactivity) at the 0–240 s time window (r = 0.556, *p* = 0.017). Additionally, HbO levels during the same interval were positively associated with anxiety scores (SCARED) (r = 0.494, *p* = 0.032).

In order to assess whether these associations were driven by influential data points, Cook’s distance and Kendall’s τ were examined. For the association between post-intervention HbO levels and SNAP-IV Hyperactivity scores, one influential observation was identified in the 0–240 s time window (Cook’s D = 0.253). After exclusion of this observation, the association no longer reached significance but rather showed a trend-level effect (Spearman r = 0.473, *p* = 0.055). In the full sample, Kendall’s τ was significant for the 0–240 s time window (τ = 0.401, *p* = 0.024), leading to partial support for the robustness of this association.

For the association between post-intervention HbO levels and SCARED anxiety scores, one participant was identified as highly influential at the 0–240 s time window (Cook’s D = 3.740). After excluding this participant, the correlation was no longer significant (Spearman r = 0.388, *p* = 0.123, Kendall τ = 0.299, *p* = 0.107), suggesting that the finding was substantially driven by a single data point. Therefore, the association between the HbO levels and SCARED scores should be interpreted with considerable caution and may not reflect a robust relationship in the current sample.

## 4. Discussion

This exploratory study examined the immediate effects of a single session of ACT on resting-state prefrontal hemodynamic activity in children with ADHD using fNIRS. To our knowledge, this is the first fNIRS study to investigate the resting-state hemodynamic changes associated with a single-session ACT intervention targeting emotion regulation in this population. Our findings demonstrated a significant hemodynamic alteration in HbO level in the right lateral prefrontal cortex after the intervention. In addition, post-intervention HbO levels were positively associated with SNAP hyperactivity scores, suggesting a possible relationship between the observed neural responses and symptom severity. Together, these findings provide preliminary evidence that a brief ACT intervention may be associated with short-term neural changes in prefrontal hemodynamic activity in children with ADHD. Given the exploratory design and absence of a control condition, the functional significance of these findings remains uncertain. Nevertheless, the observed changes highlight the potential utility of resting-state fNIRS for intervention-related responses in pediatric ADHD populations.

While the neural mechanisms of ADHD have been frequently examined using task-based paradigms [90,91], relatively few studies have investigated the resting-state hemodynamic activity. Prior research suggests reduced intrinsic activity within the default mode network in children with ADHD [52,92]. In the present study, we used resting-state fNIRS to examine whether a single-session ACT intervention would change baseline neural activity related to emotion regulation and cognitive control. Our findings indicated increased HbO in the right lateral prefrontal cortex following the ACT session, suggesting measurable changes in resting-state hemodynamics following the intervention. The lateral PFC, a key component of the frontoparietal control network, is implicated in top-down regulation of emotional responses and cognitive control. This pattern is broadly consistent with the ACT framework, which emphasizes psychological flexibility through nonjudgmental awareness and acceptance of internal experiences rather than directly controlling or suppressing them. Overall, these findings suggest that prefrontal hemodynamic activity in children with ADHD may show changes even following a brief ACT intervention, pointing to early neural changes that may precede observable clinical effects.

Of particular importance, our study evaluated the temporal stability of these changes using a time window analysis approach. To address this, we examined both the magnitude (i.e., the level of hemodynamic activity) and temporal stability (i.e., consistency of this activity over time) of changes in fNIRS. While the optimal window length for time-window analyses remains debated [93], we used time intervals of 2, 4, and 8 min across the total 8-min resting-state recording in line with previous studies that suggested that at least 2.5 min is required to obtain a stable and trustworthy functional connectivity [94,95]. In the present study, increased HbO levels in the right lateral PFC were most detectable in the 4-min window. Importantly, these findings suggest that post-intervention hemodynamic changes may be observed within intermediate-duration resting-state intervals, highlighting the importance of considering temporal stability when evaluating fNIRS-derived neural responses following behavioral interventions.

Post-intervention findings revealed that increased prefrontal hemodynamic responses were positively associated with SNAP hyperactivity scores, suggesting that individuals with greater hyperactivity showed higher levels of cortical activation after the ACT intervention. In parallel, the baseline negative association between psychological flexibility and right lateral prefrontal activation suggests that lower flexibility may be associated with increased engagement of lateral PFC, a region implicated in cognitive control and regulatory processing. Taken together, this pattern may be consistent with compensatory recruitment of prefrontal resources, as has been reported in ADHD populations, potentially reflecting increased effort during neural processing. However, given the exploratory design, these interpretations remain tentative and require replication in larger controlled samples.

In the present study, fNIRS data were successfully acquired from all participants, with no dropouts or exclusions due to intolerance or non-compliance. The procedure was well tolerated in the pediatric ADHD group, with no adverse effects reported and full completion of the protocol. As fNIRS has increasingly been used as a feasible and well tolerated method for capturing hemodynamic changes following interventions [96,97,98,99,100,101,102,103,104], our findings support the feasibility and acceptability of fNIRS in children with ADHD, a population in which neuroimaging techniques such as fMRI are often limited by motion sensitivity, intolerance, and high exclusion rates [96]. Prior task-based studies have similarly demonstrated the feasibility and reliability of fNIRS in this population, particularly in relation to attention and executive function tasks [52,96,99,100,101,102]. Importantly, the present findings extend this literature by supporting the utility of fNIRS for detecting acute and potentially subtle intervention-related changes in cortical hemodynamics. Taken together, our results provide support that fNIRS may be a well tolerated and methodologically robust neuroimaging tool for investigating both task and intervention-related hemodynamic alterations in children with ADHD.

To our knowledge, this is the first fNIRS study measuring the immediate neural effects of a single-session psychological intervention targeting emotion regulation in individuals with ADHD at rest. As a preliminary study, several limitations should be acknowledged. First, the absence of a control group limits our ability to determine whether the observed hemodynamic changes reflect normalization toward typical activation patterns or are specific to the ADHD population. However, the within-subject pre–post design allows for the detection of immediate changes in neural responses, independent of between-group variability, and provides preliminary evidence of changes in prefrontal hemodynamic activity following ACT. Secondly, the study did not include a direct assessment of behavioral outcomes following the single-session intervention. Given the single-session nature of the intervention, measurable behavioral changes may not be expected to emerge immediately, whereas neural responses may provide a more sensitive index of early intervention effects. Accordingly, this study adopted an exploratory, mechanistic approach focusing on the immediate neurophysiological correlates of ACT, as such changes may be detectable at the neural level before translating into observable behavioral improvements. Third, the relatively small sample size limits the generalizability of the findings. Given the small sample size, subgroup analyses (including sex/gender-related heterogeneity in fNIRS responses) were not feasible; however, this variability should be considered when interpreting the findings. Also, the clinical sample was heterogeneous in terms of symptom profiles, and subtype-specific differences were not examined. Future studies with larger and more demographically diverse samples may benefit from demography-aware modeling approaches to improve subgroup generalizability and fairness in clinical prediction models [103]. Finally, variability in medication history represents a potential confounding factor. Although participants were not under the acute effects of methylphenidate at the time of fNIRS acquisition, all had a history of sustained treatment. The long-term effects of methylphenidate on cerebral hemodynamics remain insufficiently understood and inconsistently reported, and the lack of detailed stratification by treatment duration and cumulative dose further limits interpretation.

In recent years, there has been growing interest in brief and single-session ACT interventions, which aim to deliver core therapeutic processes in a time-efficient and accessible format [48]. Such interventions are particularly valuable in children and adolescents [104], where engagement, time, and service accessibility may be limited [48,105]. For children with ADHD, a single-session ACT approach may provide a practical and scalable method to introduce coping skills and enhance self-regulatory processes. Accordingly, the incorporation of brief ACT interventions into ADHD treatment frameworks may offer considerable potential, both as an independent form of support and as an adjunct to existing behavioral and pharmacological approaches, although further evidence is needed to establish their clinical utility. Findings from this preliminary study suggest that single-session ACT may be followed by measurable changes in neural activity in children with ADHD; however, the functional significance of these changes currently remains unclear. Future studies with larger, more homogeneous samples and control groups, and longitudinal behavioral assessments are needed to clarify the clinical relevance of these findings and to further explore whether such neural changes are associated with improvements in ACT-related outcomes.

## 5. Conclusions

In conclusion, the present findings provide preliminary evidence that a single-session ACT intervention may be associated with short-term, measurable changes in prefrontal hemodynamic activity in children with ADHD. Specifically, increased HbO levels were observed in the right lateral prefrontal cortex after the intervention, although the functional significance of this finding remains to be clarified in future studies. These findings highlight the sensitivity of fNIRS for detecting rapid, subtle hemodynamic changes in pediatric populations. Given the exploratory nature of the study, further research with larger samples, control groups, and longitudinal behavioral outcomes is needed to evaluate the reproducibility of these findings and their clinical relevance.

## Figures and Tables

**Table 1:** ACT-based protocol with core processes, aims, and practice examples.

Core Process	Aim	Practice Examples
Acceptance	Identify and increase awareness of emotions Help to normalize anxiety-provoking function of the mind and increase awareness and acceptance of internal experiences (uncomfortable thoughts and feelings) Promote defusion and self-compassion by highlighting out of control-working mechanism of mind	Visual emotion cards: describe when you feel like this (triggers of the emotion) Caveman mind metaphor: discuss the mind’s functions. Psychologist aims to clarify mind’s natural function of creating disturbing thoughts and feelings, that are beyond our control, even in the absence of a real danger because of evolutionary heritage
Mindfulness	Notice shift of mind between past and future-oriented thoughts, and foster present moment-awareness	Jelly snake exercise: focus on five sensory properties
Values	Identify personal values in life domains	Values clarification exercise (inspired by the Eulogy exercise) and Quick Values Checklist: Imagine you are going to receive a lifetime achievement award. Check off items on the list indicating how you would like to be announced on stage at the awards ceremony
Committed actions, Acceptance	Accept disturbing internal experiences and engage in committed actions for a meaningful life	Quick Values Checklist: Discuss sometimes it is not easy to live in accordance with personal values referred to this list: The Winding and Rocky Road of Values: Dealing with Hooks exercise [77].

**Table 2:** Demographic and clinical characteristics by ADHD subtype.

Characteristics	Inattentive (n = 7) Mean ± SD	Combined (n = 13) Mean ± SD	Total (n = 20) Mean ± SD
ADHD Subtype, n (%)	7 (35%)	13 (65%)	20 (100%)
Gender (F/M)	4/3	6/7	10/10
Age	11.2 ± 1.3	10.9 ± 1.4	11.1 ± 1.4
ACT-Related measures			
CAMM	24.00 ± 5.59	23.08 ± 5.35	23.37 ± 5.28
AFQ	6.00 ± 4.20	5.09 ± 3.05	5.41 ± 3.39
Clinical measures			
WFIRS	0.69 ± 0.48	1.13 ± 0.52	0.98 ± 0.53
CDI	24.67 ± 3.07	23.92 ± 7.47	24.16 ± 6.32
CGI	4.43 ± 0.54	4.54 ± 0.78	4.50 ± 0.69
Parent scales			
SNAP-IV attention	1.96 ± 0.39	1.54 ± 0.46	1.68 ± 0.47
SNAP-IV hyperactivity	0.91 ± 0.88	1.43 ± 0.53	1.26 ± 0.69
SCARED	20.83 ± 11.82	23.85 ± 18.56	22.89 ± 16.45

ADHD = Attention Deficit Hyperactivity Disorder; CAMM = Child and Adolescent Mindfulness Measure; AFQ = Acceptance and Fusion Questionnaire; WFIRS = Weiss Functional Impairment Rating Scale; CDI = Children’s Depression Inventory; CGI = Clinical Global Impression; SNAP-IV = Swanson, Nolan, and Pelham Rating Scale; SCARED = Screen for Child Anxiety Related Emotional Disorders; SD = standard deviation.

**Table 3:** Bonferroni-corrected and raw significance scores of paired sample *t*-test comparisons between after-before conditions along hemodynamic parameters. Only significant time interval results are listed.

Signal	Metric	Time Interval	Channel	N	Mean Difference (After- Before)	SD	t	Cohen’s d	Cohen’d Effect Size	Bonferroni Alpha (0.05/n = 3)	*p* Two-Sided	*p* Bonferroni Corrected
HbO	Range	0_240	Right Lateral PFC	20	0.000021	0.000034	2.73266	0.611	Medium	0.016667	* **0.0132**	* **0.039667**

HbO = Oxygenated hemoglobin; PFC = prefrontal cortex; SD = standard deviation; Bold font and * indicate *p* < 0.05.

## Data Availability

The data presented in this study are available upon reasonable request from the corresponding author. The data are not publicly available due to ethical restrictions.

## Supplementary Materials

The following supporting information can be downloaded at: https://www.mdpi.com/article/10.3390/brainsci16060564/s1, Table S1: complete statistical results.

## Abbreviations

The following abbreviations are used in this manuscript:

ADHD	Attention Deficit Hyperactivity Disorder
fNIRS	Functional Near-Infrared Spectroscopy
ACT	Acceptance and Commitment Therapy
PFC	Prefrontal Cortex
HbO	Oxyhemoglobin
HbR	Deoxyhemoglobin
