# Supplementary material for: Immediate Effects of Acceptance and Commitment Therapy in Children with ADHD: A Pilot Resting-State fNIRS Study
Source: Brain Sci. 2026 May 27;16(6):564. doi: 10.3390/brainsci16060564 (PMC13297458; doi:10.3390/brainsci16060564)
Supplement: Supplementary file 1 [file brainsci-16-00564-s001.zip › brainsci-4305388-supplementary.pdf]

Chromophore	Metric	Time Interval	Channel	N	Mean_Diff(After-Before)	Std_Deviation	Std_Error_Mean	95%CI_Lower	95%CI_Upper	t	df	p_OneSided	p_TwoSided	Cohens_d	Cohens_d Effect_Size	N_Comparisons (Bonferroni)	Bonferroni_Alpha(0.05/n=3)	p_Bonferroni	Significance Status
HbO	RANGE	CUM_0_120	RightLat	20	0,000006	0,000024	0,000005	-0,000005	0,000018	1,211152	19	0,120343	0,240687	0,270822	small	3	0,016667	0,722061	
HbO	RANGE	CUM_0_120	Medial	20	-0,000057	0,000519	0,000116	-0,0003	0,000186	-0,492377	19	0,314047	0,628094	-0,110099	negligible	3	0,016667	1	
HbO	RANGE	CUM_0_120	LeftLat	20	-0,000032	0,000304	0,000068	-0,000174	0,000111	-0,467624	19	0,322686	0,645373	-0,104564	negligible	3	0,016667	1	
HbO	RANGE	CUM_0_240	RightLat	20	0,000021	0,000034	0,000008	0,000005	0,000037	2,732663	19	0,006611	0,013222	0,611042	medium	3	0,016667	0,039667	Bonferroni significant
HbO	RANGE	CUM_0_240	Medial	20	-0,000044	0,000525	0,000117	-0,00029	0,000201	-0,37742	19	0,355022	0,710045	-0,084394	negligible	3	0,016667	1	
HbO	RANGE	CUM_0_240	LeftLat	20	-0,000011	0,000338	0,000076	-0,000169	0,000147	-0,142174	19	0,44422	0,888439	-0,031791	negligible	3	0,016667	1	
HbO	RANGE	CUM_0_480	RightLat	20	0,000019	0,000034	0,000007	0,000003	0,000035	2,536827	19	0,010056	0,020113	0,567252	medium	3	0,016667	0,060339	uncorrected significant
HbO	RANGE	CUM_0_480	Medial	20	0,000002	0,000585	0,000131	-0,000272	0,000275	0,013773	19	0,494577	0,989154	0,00308	negligible	3	0,016667	1	
HbO	RANGE	CUM_0_480	LeftLat	20	0,000012	0,000354	0,000079	-0,000154	0,000177	0,145336	19	0,442988	0,885977	0,032498	negligible	3	0,016667	1	
HbO	STD	CUM_0_120	RightLat	20	0,000002	0,000005	0,000001	-0,000001	0,000004	1,383787	19	0,091233	0,182466	0,309424	small	3	0,016667	0,547398	
HbO	STD	CUM_0_120	Medial	20	-0,000016	0,000129	0,000029	-0,000077	0,000044	-0,570257	19	0,287593	0,575185	-0,127513	negligible	3	0,016667	1	
HbO	STD	CUM_0_120	LeftLat	20	-0,000004	0,000066	0,000015	-0,000035	0,000026	-0,288474	19	0,388054	0,776107	-0,064505	negligible	3	0,016667	1	
HbO	STD	CUM_0_240	RightLat	20	0,000003	0,000006	0,000001	0	0,000006	2,411476	19	0,013086	0,026172	0,539223	medium	3	0,016667	0,078517	uncorrected significant
HbO	STD	CUM_0_240	Medial	20	-0,000006	0,000096	0,000022	-0,000051	0,000039	-0,263448	19	0,397521	0,795041	-0,058909	negligible	3	0,016667	1	
HbO	STD	CUM_0_240	LeftLat	20	-0,000002	0,000064	0,000014	-0,000032	0,000028	-0,152059	19	0,440372	0,880743	-0,034001	negligible	3	0,016667	1	
HbO	STD	CUM_0_480	RightLat	20	0,000003	0,000005	0,000001	0	0,000005	2,332533	19	0,015411	0,030822	0,52157	medium	3	0,016667	0,092466	uncorrected significant
HbO	STD	CUM_0_480	Medial	20	0,000004	0,000082	0,000018	-0,000034	0,000042	0,220259	19	0,414009	0,828019	0,049251	negligible	3	0,016667	1	
HbO	STD	CUM_0_480	LeftLat	20	0,000001	0,000061	0,000014	-0,000028	0,000029	0,061641	19	0,475746	0,951493	0,013783	negligible	3	0,016667	1	
HbR	RANGE	CUM_0_120	RightLat	20	0,000251	0,001004	0,000225	-0,000219	0,000721	1,116602	19	0,139044	0,278087	0,24968	small	3	0,016667	0,834261	
HbR	RANGE	CUM_0_120	Medial	20	0,00014	0,002251	0,000503	-0,000914	0,001193	0,277113	19	0,392343	0,784686	0,061964	negligible	3	0,016667	1	
HbR	RANGE	CUM_0_120	LeftLat	20	-0,000014	0,006662	0,00149	-0,003132	0,003104	-0,009501	19	0,496259	0,992518	-0,002125	negligible	3	0,016667	1	
HbR	RANGE	CUM_0_240	RightLat	20	0,000464	0,001172	0,000262	-0,000085	0,001012	1,770325	19	0,046359	0,092718	0,395857	small	3	0,016667	0,278153	
HbR	RANGE	CUM_0_240	Medial	20	0,000287	0,002406	0,000538	-0,000839	0,001413	0,533183	19	0,300044	0,600089	0,119223	negligible	3	0,016667	1	
HbR	RANGE	CUM_0_240	LeftLat	20	0,000439	0,007103	0,001588	-0,002885	0,003764	0,276628	19	0,392526	0,785053	0,061856	negligible	3	0,016667	1	
HbR	RANGE	CUM_0_480	RightLat	20	0,000467	0,001289	0,000288	-0,000137	0,00107	1,618985	19	0,060965	0,121931	0,362016	small	3	0,016667	0,365792	
HbR	RANGE	CUM_0_480	Medial	20	0,000138	0,003065	0,000685	-0,001297	0,001572	0,200873	19	0,421466	0,842933	0,044917	negligible	3	0,016667	1	
HbR	RANGE	CUM_0_480	LeftLat	20	-0,000297	0,008098	0,001811	-0,004087	0,003492	-0,164193	19	0,435657	0,871313	-0,036715	negligible	3	0,016667	1	
HbR	STD	CUM_0_120	RightLat	20	0,000056	0,000243	0,000054	-0,000058	0,000169	1,031149	19	0,15771	0,315419	0,230572	small	3	0,016667	0,946257	
HbR	STD	CUM_0_120	Medial	20	0,000032	0,000588	0,000131	-0,000243	0,000307	0,243331	19	0,405178	0,810356	0,054411	negligible	3	0,016667	1	
HbR	STD	CUM_0_120	LeftLat	20	0,000054	0,001634	0,000365	-0,000711	0,000819	0,14755	19	0,442126	0,884252	0,032993	negligible	3	0,016667	1	
HbR	STD	CUM_0_240	RightLat	20	0,000076	0,000198	0,000044	-0,000017	0,000168	1,718825	19	0,050949	0,101898	0,384341	small	3	0,016667	0,305693	
HbR	STD	CUM_0_240	Medial	20	0,000057	0,00046	0,000103	-0,000158	0,000273	0,554809	19	0,292749	0,585498	0,124059	negligible	3	0,016667	1	
HbR	STD	CUM_0_240	LeftLat	20	0,00012	0,001391	0,000311	-0,000531	0,000772	0,386938	19	0,351551	0,703102	0,086522	negligible	3	0,016667	1	
HbR	STD	CUM_0_480	RightLat	20	0,000052	0,000175	0,000039	-0,00003	0,000134	1,331473	19	0,099394	0,198789	0,297726	small	3	0,016667	0,596366	
HbR	STD	CUM_0_480	Medial	20	0,000051	0,000425	0,000095	-0,000147	0,00025	0,54051	19	0,297563	0,595126	0,120862	negligible	3	0,016667	1	
HbR	STD	CUM_0_480	LeftLat	20	-0,000023	0,001171	0,000262	-0,000571	0,000525	-0,087267	19	0,465686	0,931372	-0,019514	negligible	3	0,016667	1	
HbT	RANGE	CUM_0_120	RightLat	20	0,00025	0,001005	0,000225	-0,00022	0,000721	1,113435	19	0,139705	0,27941	0,248972	small	3	0,016667	0,838231	
HbT	RANGE	CUM_0_120	Medial	20	0,000103	0,002226	0,000498	-0,000939	0,001145	0,207559	19	0,418891	0,837781	0,046412	negligible	3	0,016667	1	
HbT	RANGE	CUM_0_120	LeftLat	20	0,000047	0,006674	0,001492	-0,003077	0,003171	0,031539	19	0,487584	0,975169	0,007052	negligible	3	0,016667	1	
HbT	RANGE	CUM_0_240	RightLat	20	0,000478	0,001167	0,000261	-0,000068	0,001024	1,830792	19	0,041431	0,082862	0,409377	small	3	0,016667	0,248587	
HbT	RANGE	CUM_0_240	Medial	20	0,000248	0,002376	0,000531	-0,000863	0,00136	0,467763	19	0,322637	0,645275	0,104595	negligible	3	0,016667	1	
HbT	RANGE	CUM_0_240	LeftLat	20	0,000482	0,007089	0,001585	-0,002836	0,0038	0,304032	19	0,382204	0,764408	0,067984	negligible	3	0,016667	1	
HbT	RANGE	CUM_0_480	RightLat	20	0,000474	0,001281	0,000287	-0,000126	0,001073	1,653393	19	0,057339	0,114679	0,36971	small	3	0,016667	0,344037	
HbT	RANGE	CUM_0_480	Medial	20	0,000124	0,003033	0,000678	-0,001296	0,001544	0,182969	19	0,42838	0,856761	0,040913	negligible	3	0,016667	1	
HbT	RANGE	CUM_0_480	LeftLat	20	-0,000297	0,008092	0,001809	-0,004084	0,00349	-0,164393	19	0,435579	0,871158	-0,036759	negligible	3	0,016667	1	
HbT	STD	CUM_0_120	RightLat	20	0,000055	0,000243	0,000054	-0,000059	0,000169	1,015331	19	0,161352	0,322704	0,227035	small	3	0,016667	0,968112	
HbT	STD	CUM_0_120	Medial	20	0,000018	0,000571	0,000128	-0,000249	0,000285	0,138118	19	0,4458	0,8916	0,030884	negligible	3	0,016667	1	
HbT	STD	CUM_0_120	LeftLat	20	0,000065	0,001625	0,000363	-0,000696	0,000825	0,178426	19	0,430138	0,860277	0,039897	negligible	3	0,016667	1	
HbT	STD	CUM_0_240	RightLat	20	0,000077	0,000197	0,000044	-0,000015	0,00017	1,754007	19	0,047773	0,095546	0,392208	small	3	0,016667	0,286637	
HbT	STD	CUM_0_240	Medial	20	0,000046	0,000448	0,0001	-0,000164	0,000256	0,460049	19	0,325351	0,650702	0,10287	negligible	3	0,016667	1	
HbT	STD	CUM_0_240	LeftLat	20	0,000123	0,001385	0,00031	-0,000526	0,000771	0,395562	19	0,348417	0,696834	0,08845	negligible	3	0,016667	1	
HbT	STD	CUM_0_480	LeftLat	20	-0,000028	0,001166	0,000261	-0,000573	0,000518	-0,106155	19	0,458286	0,916572	-0,023737	negligible	3	0,016667	1	
